# Stimulation of *Mytilus galloprovincialis* Hemocytes With Different Immune Challenges Induces Differential Transcriptomic, miRNomic, and Functional Responses

**DOI:** 10.3389/fimmu.2020.606102

**Published:** 2020-12-17

**Authors:** Rebeca Moreira, Alejandro Romero, Magalí Rey-Campos, Patricia Pereiro, Umberto Rosani, Beatriz Novoa, Antonio Figueras

**Affiliations:** ^1^ Institute of Marine Research (IIM), Spanish National Research Council (CSIC), Vigo, Spain; ^2^ Department of Biology, University of Padova, Padova, Italy; ^3^ Helmholtz Centre for Polar and Marine Research, Alfred Wegener Institute (AWI), List auf Sylt, Germany

**Keywords:** *Mytilus galloprovincialis*, hemocytes, pathogen-associated molecular patterns, transcriptome, miRNA, immunity, immunometabolism

## Abstract

Mediterranean mussels (*Mytilus galloprovincialis*) are marine bivalve molluscs with high resilience to biotic and abiotic stress. This resilience is one of the reasons why this species is such an interesting model for studying processes such as the immune response. In this work, we stimulated mussel hemocytes with poly I:C, β-glucans, and LPS and then sequenced hemocyte mRNAs (transcriptome) and microRNAs (miRNome) to investigate the molecular basis of the innate immune responses against these pathogen-associated molecular patterns (PAMPs). An immune transcriptome comprising 219,765 transcripts and an overview of the mussel miRNome based on 5,175,567 non-redundant miRNA reads were obtained. The expression analyses showed opposite results in the transcriptome and miRNome; LPS was the stimulus that triggered the highest transcriptomic response, with 648 differentially expressed genes (DEGs), while poly I:C was the stimulus that triggered the highest miRNA response, with 240 DE miRNAs. Our results reveal a powerful immune response to LPS as well as activation of certain immunometabolism- and ageing/senescence-related processes in response to all the immune challenges. Poly I:C exhibited powerful stimulating properties in mussels, since it triggered the highest miRNomic response and modulated important genes related to energy demand; these effects could be related to the stronger activation of these hemocytes (increased phagocytosis, increased NO synthesis, and increased velocity and accumulated distance). The transcriptome results suggest that after LPS stimulation, pathogen recognition, homeostasis and cell survival processes were activated, and phagocytosis was induced by LPS. β-glucans elicited a response related to cholesterol metabolism, which is important during the immune response, and it was the only stimulus that induced the synthesis of ROS. These results suggest a specific and distinct response of hemocytes to each stimulus from a transcriptomic, miRNomic, and functional point of view.

## Introduction

After infection with a pathogen, vertebrates produce antibodies to protect themselves against future encounters with the same pathogen, but invertebrates rely only on their innate immune system to respond to potential infections. Bivalves do not possess acquired immunity, but after encountering microbes as part of their filter-feeder lifestyle, they can respond to infection through their defense cells, the hemocytes, with a certain degree of pathogen specificity (1–4). Similarly, immunostimulants, such as polyinosinic:polycytidylic acid (poly I:C), can enhance the defense capacities of bivalves and promote their protection against pathogens that cause high mortality; this phenomenon was mainly studied in *Crassostrea gigas* (5, 6).

In vertebrates, the innate immune response is triggered by the recognition of pathogen-associated molecular patterns (PAMPs) through pattern recognition receptors (PRRs) that can detect extracellular and intracellular non–self-molecules. The complete response pathways from PAMP detection to molecular effector production are well-studied in mammals (7, 8). For example, the viral mimic molecule poly I:C, an analogue of double-stranded RNA, is detected intracellularly by toll-like receptor 3 (TLR3), RIG-I-like receptors (RLRs) and protein kinase R (PKR); recognition of poly I:C promotes the transcription of proinflammatory cytokines, interferon (IFN) and IFN-inducible genes through nuclear factor-kappa B (NF-kB) and IFN regulatory factors (IRF) 3 and 7. β-glucans, components of the fungal cell wall, are phagocytosed *via* a mannose-binding lectin (dectin-1), and subsequently, TLR2 triggers NF-kB activation to produce proinflammatory cytokines. Lipopolysaccharides (LPS) from gram-negative bacteria are recognized by the extracellular LPS-binding protein (LBP) and then bind to CD14 on the cell surface, which allows the detection of LPS by TLR4 through the accessory molecule myeloid differentiation 2 (MD2). Next, NF-kB and IRF3 translocate to the nucleus to transcribe proinflammatory cytokines, IFN, and IFN-inducible genes.

Contrary to that observed in vertebrates, in insect the Toll dependent response is activated by the interaction with the ligand spaetzle, which is activated by proteolytic cleavage after the recognition of the PAMPs by extracellular PRRs (9). Active spaetzle bind to the Toll receptor and activates a signaling pathway that results in the phosphorylation and degradation of Cactus and the translocation of the transcription factor Relish (equivalent to NF-kB) to the nucleus to activate the transcription of effector immune genes (9). However, this seems not to be conserved for all the arthropods, since it has been shown that shrimp Tolls directly bind to PAMPs (10), as it also occurs with mollusc TLRs (11, 12). A spaetzle homolog gene has not yet been described in non-arthropod animals, with the exception of the marine mollusc, *Paphia undulate* (13). However, although its overexpression after stimulation with heat-killed bacteria has been observed, its involvement in the TLR signaling pathway has not been demonstrated (13).

In mussels, several key immune genes have been identified and characterized over the past few years. The richness of antimicrobial peptides presents in Mediterranean mussel seems to be one of the main immune properties of this bivalve species, and it has been considered one of the main factors explaining the resistance of this species to infectious diseases (14). However, a great variety of potential PRRs has been also identified in this mussel species. Gerdol and Venier (15) collected all the available information deposited in public databases to review and explore the diversity of the potential extracellular, membrane-bound and intracellular PRRs in *M. galloprovincialis*, which included C1q domain-containing (C1qDC) proteins, C-, R-, and F-type lectins, galectins, fibrinogen-related proteins (FREPs), Gram-negative binding proteins (GNBPs), apextrin-related proteins, complement components, thioester-containing proteins (TEPs), TLRs, peptidoglycan recognition receptors (PGRPs), scavenger receptor cysteine-rich superfamily (SRCR-SF) receptors, Nod-like receptors (NLRs), or RLRs.

Despite all this available information, and although PAMPs have already been used as immunostimulants in bivalves (16, 17) and some of the relevant genes have been identified (18), the molecular mechanisms underlying the immune responses to different PAMPs in this animal group are still incompletely understood. Costa et al. (19) studied the effect of different PAMPs (poly I:C, zymosan, LPS, unmethylated cytosine-guanidine motifs –CpG– and lipoteichoic acid –LTA–) on *Mytilus galloprovincialis* hemocytes; the authors observed that zymosan was the only stimulus that increased the respiratory burst activity, but LPS was the PAMP that induced the higher overexpression of the tested immune genes (myticin C, mytilin B, and lysozyme). The *in vitro* stimulation of Mediterranean mussel hemocytes with these PAMPs also revealed their effect on the expression of the apoptosis-related genes caspases (20) and FREPs (21), which are probably involved in pathogen recognition, among other functions. However, a high-throughput analysis of the transcriptome response of *M. galloprovincialis* hemocytes to different PAMPs was not previously conducted. Therefore, the primary objective of this work was to study the transcriptomic and miRNomic profiles of *M. galloprovincialis* hemocytes after immune challenge *in vitro* in order to obtain new insights into the molecular basis of the responses to poly I:C, β-glucans and LPS. To the best of our knowledge, this is the first combined transcriptomic and miRNomic study in molluscs after immune stimulation. Moreover, different aspects associated with activation (proliferation, phagocytosis, reactive oxygen species and nitric oxide synthesis, cell morphology and movement characteristics) were analyzed.

The interaction between the transcriptome and miRNome influences cellular function. miRNAs are small noncoding RNAs that are evolutionarily conserved, and they regulate gene expression at the posttranscriptional level by interacting with the 3’UTR of mRNAs and recruiting molecular machinery that degrades the target mRNAs (22). Research on miRNAs is currently of great importance in species ranging from plants (23) to humans, especially in the fields of ontogeny (24), cancer (25), and inflammation (26, 27). In recent years, many studies have contributed to the discovery of novel miRNAs; although these works were intended to shed light on the regulatory roles of these molecules, the confirmation of miRNA-mRNA target pairs has mainly been accomplished based upon interaction or expression patterns but not functional targeting (28). Despite this limitation, studies conducted in different organisms can provide valuable information about the mechanisms of action of these molecules because evolutionary studies have shown that miRNA families have been continuously added along bilaterian evolution while loss events appear to be rare and that mature miRNA sequences are conserved under strict negative selection (29, 30). In bivalves, the role of miRNAs is an almost unexplored field, and only a handful of research studies on this topic have been published in the last 5 years. Rosani et al. (31) recently described the mechanism underlying the generation of miRNAs in mussels and oysters. The role of miRNA has also been investigated in the bivalve immune response (32), neuroendocrine system (33), osmotic stress (34), or metamorphosis (35).

Our goal was to study the differential expression of mRNAs and miRNAs in mussel hemocytes stimulated *in vitro* with specific PAMPs and to determine how the miRNome is related to the transcriptomic immune responses of mussels. Moreover, the functional aspects of the stimulated hemocytes were analyzed to better understand the biological significance of these alterations.

## Materials and Methods

### Animals

Adult *M. galloprovincialis*, 8-10 cm in shell length, were obtained from a commercial shellfish farm (Vigo, Galicia, Spain) and maintained in open-circuit filtered seawater tanks at 15°C with aeration. The mussels were fed daily with *Phaeodactylum tricornutum* and *Isochrysis galbana*. Prior to the experiments, the animals were allowed to acclimate to the aquarium conditions for at least 1 week.

### Experimental Design, RNA Isolation, and Illumina Sequencing of mRNAs and miRNAs

Mussels were notched on the shell, and hemolymph (1 ml) was withdrawn from the adductor muscle with a 0.5-mm diameter (25-G) disposable needle. The hemolymph from 25 individuals was pooled and distributed into a 6-well plate in a volume of 5 ml per well in a total of 4 wells, that is, one well for each treatment. The hemocytes were allowed to settle to the base of the wells for 30 min at 15°C in the dark. Then, the hemocytes were stimulated for 8 h at 15°C with 50 μg/ml of poly I:C, β-glucans, and LPS. The last group of hemocytes remained unstimulated. All the stimuli were purchased from Sigma Aldrich (St. Louis, MO, USA). This procedure was performed in triplicate. After 8 h of stimulation, the hemolymph was recovered from each well and centrifuged (4°C, 3000 ×g, 10 min). The pellet was resuspended in 300 μl of homogenization buffer (Maxwell simply RNA purification kits AS1270, Promega, Madison, WI, USA) and immediately homogenized with a 1 ml syringe equipped with a 25-G needle. RNA was isolated from the 12 samples using the Maxwell LEV robot following the instructions of the Maxwell simply RNA tissue kit (Promega). Next, the concentration and purity of the RNA were measured using a NanoDrop ND1000 spectrophotometer (NanoDrop Technologies Inc., DE, USA), and the RNA integrity was tested on an Agilent 2100 Bioanalyzer (Agilent Technologies, CA, USA) before producing the libraries for Illumina sequencing. Both mRNA and small RNA library preparation and next-generation sequencing were performed at Macrogen (Seoul, Republic of Korea).

For mRNA, the TruSeq Stranded mRNA LT Sample Preparation Kit from Illumina (San Diego, CA, USA) was used according to the manufacturer’s instructions. mRNA was extracted from total RNA using oligo (dT) magnetic beads and cleaved into short fragments using fragmentation buffer. A cDNA library compatible with Illumina NGS technology was then prepared from the fragmented mRNA *via* reverse transcription, second-strand synthesis and ligation of specific adapters (paired-ends) after cDNA purification using the QIAquick PCR Purification Kit (Qiagen, Hilden, Germany). The amount of cDNA in each library was quantified through spectrofluorometric analysis using the Qubit system. The paired-end 100 bp (PE100) data were obtained by Illumina HiSeq™ 4000 technology.

For small RNAs, the TruSeq small RNA Library Preparation Kit from Illumina (San Diego, CA, USA) was used according to the manufacturer’s instructions. Briefly, small RNAs were specifically ligated to 5′ and 3′ adapters, reverse transcribed and amplified and indexed by PCR. The purified pooled PCR products were used to generate the library product. The amount of cDNA in each library was quantified by Qubit, and single-end 50-bp (SE50) sequencing was performed using Illumina HiSeq™ 2500 technology.

### Bioinformatics and RNA-Seq Analyses

CLC Genomics Workbench v.12 (CLC Bio, Qiagen, Hilden, Germany) was used for filtering, assembly, RNA-Seq and statistical analyses of both the transcriptome and miRNome. In both cases, the raw reads were trimmed using the modified-Mott trimming algorithm to remove low-quality sequences (Phred score limit of 0.05) and adaptor sequences. mRNA sequences shorter than 70 base pairs (bp) were also removed. For the miRNA data, reads in the 15-30 nucleotide range were selected.

A reference global transcriptome resulting from the 12 mRNA samples was *de novo* assembled through de Bruijn graph as algorithmic technique with an overlap criterion of 70% and a similarity of 90% to exclude paralogous sequence variants. The minimum transcript length was set to 200 base pairs.

For the miRNome data, all the trimmed reads were clustered together, allowing only perfect matches, to obtain a global non-redundant miRNome. The number of matching reads for each cluster was counted, and only the clusters with a minimal representation of 52 reads were further considered (reads per million > 10). The resulting putative miRNAs were annotated against miRBase v.22.1 (36), allowing a maximum of 2 mismatches and 2 missing or additional 5’ or 3’ bases.

Finally, for both the transcriptome and miRNome, RNA-Seq analysis was performed on every sample (for mRNA, the following parameters were used: length fraction = 0.8, similarity fraction = 0.8, and maximum hits per read = 10; for miRNA, the following parameters were used: length and similarity fraction = 1). The expression values were set to transcripts per million (TPM). A differential expression analysis test (Robinson and Smyth’s Exact Test, which assumes a negative binomial distribution of the data and takes into account the overdispersion caused by biological variability) was used to compare the expression levels in each sample and to identify the differentially expressed genes (DEGs) or differentially expressed miRNAs (DE miRNAs). Sequences with absolute fold change (FC) values > 2 and false discovery rate (FDR) corrected p-value < 0.05 were retained for further analyses. In the case of DE miRNAs, only mature miRNAs were chosen.

### BLAST Annotation, GO Assignment, Enrichment, and KEGG Analysis

UniProt/SwissProt BLASTx results were used to obtain the Gene Ontology (GO) term assignments of the transcript list with Blast2GO software (37). To improve the percentage of identification of the mussel transcripts, they were also annotated using BLASTn against a custom database that includes all of the molluscan sequences available in the NBCI nucleotide database. In both blast approaches, the e-value threshold was set to 1x10e-3. Then, enrichment analyses of the up- and downregulated DEGs (test set) were conducted, and these analyses included the global mussel transcriptome as the reference set. Fisher’s exact test was performed with default values and a p-value cut-off of 0.05. The option to reduce the enriched list to the most specific GO terms was used. Only overrepresented biological process (BP) GO terms were further analyzed.

The pathways in which the DEGs unique to each stimulus were involved were also analyzed. The Kyoto Encyclopedia of Genes and Genomes (KEGG) in Blast2GO was used for this purpose. A summary of the results was prepared following the existing categories in the KEGG database (http://www.genome.jp/kegg/pathway.html).

### Validation of RNA-Seq

A new experiment, performed with individual mussels, was carried out following the same design as that for the sequencing experiment (**Supplementary Figure 1A**). Briefly, the hemolymph was extracted from individual mussels and divided into four wells (250 μl per well) in a 24-well plate. Three wells were stimulated with poly I:C, β-glucans and LPS, and the last well served as the control. After 8 h, the hemolymph was recovered from each well, and RNA was extracted as previously described. cDNA was synthesized from the mRNA and miRNA of each individual mussel using 200 ng of total RNA and the NZY First-Strand cDNA Synthesis Kit (NZYtech, Lisbon, Portugal) and miScript II Retrotranscripton Kit (Qiagen, Hilden, Germany), respectively, following the manufacturers’ protocols.

Specific PCR primers were designed based on the sequences of the selected genes and miRNAs (**Supplementary Figures 1B, C** **)** using Primer3 (38) and Oligo Analyzer 1.0.2 according to qPCR restrictions. The efficiency of each primer pair was calculated prior to validation. The efficiency of each primer pair was analyzed with seven five-fold serial dilutions of cDNA and was calculated based on the slope of the regression line of the quantification cycle versus the relative cDNA concentration (39). A melting curve analysis was also performed to verify that only specific amplification occurred.

The expression of selected genes was analyzed in a 7300 Real Time PCR System (Applied Biosystems, Foster City, CA, USA). For mRNAs, 1 μl of five-fold-diluted cDNA template was mixed with 0.5 μl of each primer (10 μM) and 12.5 μl of SYBR Green PCR master mix (Applied Biosystems, Foster City, CA, USA) in a final volume of 25 μl. The standard cycling conditions were 95°C for 10 min, followed by 40 cycles of 95°C for 15 s and 60°C for 30 s. For miRNAs, the reaction was prepared following the miScript SYBR Green PCR Kit (Qiagen, Hilden, Germany) instructions (1 μl cDNA template, 2.5 μl specific miRNA primer, 2.5 μl universal primer, and 12.5 μl QuantiTec SYBR Green PCR master mix in a final volume of 25 ml). The standard cycling conditions for miRNA amplification were 95°C for 15 min, followed by 40 cycles of 95°C for 15 s, 55°C for 30 s and 70°C for 34 s. All the reactions were performed in technical triplicates.

The relative expression levels of the genes were normalized using 18S as a reference gene for mRNA (40) and 5S as a reference gene for miRNA (34) following the Pfaffl method (39). The relative expression was standardized to the normalized expression in the control hemocytes to calculate fold changes. Linear regression and correlation of log10-fold changes were performed to validate the RNA-Seq vs. qPCR results of the studied genes/miRNAs and conditions.

### Cell Viability Assays

The hemolymph was extracted from four individual mussels, diluted 1:2 in ice-cold PBS and counted at time 0 (t0). Then, the hemolymph from each individual was divided into four wells (250 μl per well) in a 24-well plate. After 30 min at 15°C to allow the hemocytes to settle, three wells (one well per mussel) were stimulated with poly I:C, β-glucans or LPS at a final concentration of 50 µg/ml. The control wells remained unstimulated. The hemocytes were completely recovered from each well by slowly pipetting up and down after 4 and 8 h of treatment with each PAMP, stained with trypan blue (1:10) and immediately counted in a Neubauer chamber to calculate the number of live and dead hemocytes. The statistical analysis (t-test, p<0.05) to identify significant differences between the control and treated samples in all these experiments was performed in GraphPad Prism (San Diego, CA, USA). This experiment was repeated twice with similar results.

### Phagocytosis

Hemolymph was collected from six pools with three mussels each, and the hemocytes were adjusted to 10^6^ cells/ml with ice-cold filtered sea water (FSW). Then, the cells were seeded into 96-well plates and incubated with the different PAMPs for 1, 4, and 8 h as previously described. To measure phagocytosis levels, we used a FACS Calibur Flow Cytometer (Becton and Dickinson, San Jose, CA, USA). A suspension of FluoSpheres (Thermo Fisher Scientific, Waltham, MA, USA) with a diameter of 1 µm (hemocytes:beads ratio of 1:100) was added, followed by incubation for 45 min at 15°C in the dark. To remove the non-phagocytosed FluoSpheres, the supernatants were removed, and the adherent cells were washed twice with FSW. The fluorescence of the stimulated hemocytes was measured. The analysis was carried out using CellQuest software (Becton and Dickinson, San Jose, CA, USA). Statistical analysis was performed in GraphPad Prism (San Diego, CA, USA) with a two-way ANOVA (p<0.05). This experiment was repeated twice with similar results.

### Respiratory Burst: Reactive Oxygen Species and Nitric Oxide Synthesis

Hemolymph was collected from three pools with three mussels each using a disposable syringe, and the cell concentration was adjusted to 10^6^ cells/ml with ice-cold FSW. Then, the cells were seeded in 96-well plates (100 µl per well). After 30 min of adherence at 15°C, the hemocytes were incubated with poly I:C, β-glucans and LPS at a final concentration of 50 µg/ml for 1, 4, and 8 h.

The ROS activity of the hemocytes was determined by the luminol-enhanced chemiluminescence method (CL) in 96-well plates. We used 5-amino-2,3-dihydro-1,4-phthalazinedione (Luminol, Sigma Aldrich, St. Louis, MO, USA) as a light emitter and phorbol myristate acetate (PMA, Sigma Aldrich, St. Louis, MO, USA) as a trigger of ROS production. A stock solution of 0.1 M luminol was prepared in dimethyl sulphoxide (DMSO, Sigma Aldrich, St. Louis, MO, USA) and diluted in FSW to obtain a luminol working solution (final concentration of 10 mM). The PMA stock solution (1 mg/ml) was diluted in the luminol working solution (final concentration of 1 µg/ml). After incubation of the cells with the different PAMPs, the PMA solution was added (100 µl per well). Then, the generation of relative luminescence units (RLU) was measured in a luminometer (Fluoroskan Ascent, LabSystems) six times at intervals of 5 min with an integration time of 1000 ms in each measurement. The statistical analysis (two-way ANOVA, p<0.05) was performed in GraphPad Prism software. This experiment was repeated twice with similar results.

NO production was assessed by quantifying the nitrite content of the supernatants. After incubation of the cells with the PAMPs, 50 μl of the supernatants were placed in a 96-well plate. A volume of 100 μl of 1% sulfanilamide (Sigma Aldrich, St. Louis, MO, USA) in 2.5% phosphoric acid was added to each well, followed by 100 μl of 0.1% N-naphthyl-ethylenediamine (Sigma Aldrich, St. Louis, MO, USA) in 2.5% phosphoric acid. The optical density (OD) was measured at 540 nm using a Multiscan Spectrophotometer (LabSystems). The µM concentration of nitrite in each sample was determined based on the standard curves generated using known concentrations of sodium nitrite. One‐way ANOVA with *post hoc* Tukey’s test was conducted using GraphPad Prism software, and the results were considered significant at a threshold p‐value < 0.05. This experiment was repeated twice with similar results.

### Live Cell Time-Lapse Microscopy and Analysis

One milliliter of hemolymph was extracted from the adductor muscle of one healthy adult mussel and immediately diluted 1:20 in ice-cold FSW to avoid cell aggregation. The mean concentration of the cells was 10^5^ cells/ml. Five hundred microliters of cells were placed in a 24-well plate and allowed to settle for 30 min at room temperature (22°C). Two parallel plates were prepared with the same hemolymph. The cells in the control plate were treated with 250 µl of FSW. The stimulated cells were treated with 250 µl of a solution containing LPS, β-glucans or poly I:C at a final concentration of 50 µg/ml. These experiments were performed 3 times.

The control plate was transferred to a TMS inverted microscope (Nikon Corp., Tokyo, Japan) equipped with phase contrast objectives and a DMX 1200 camera (Nikon Corp., Tokyo, Japan). Nikon ACT-1 v2.7 acquisition software was used to capture 8-bit digital 3840 x 3072-pixel greyscale images (1 px = 0.1366 um). The stimulated cells were imaged with a TS100 Eclipse inverted microscope (Nikon) equipped with a DS-Fi1 camera. Nis-Elements V2.32 software acquired 8-bit digital 2569x1920-pixel greyscale images (1 px = 0.33 um). Time-lapse recordings were simultaneously performed on both plates in parallel at room temperature, and images were captured every 30 s for 3 h of stimulation.

The individual images of the time-lapse sequence were processed to compute the morphological and velocimetric parameters using the Manual Tracking and the Chemotaxis and Migration Tool 2.0 plugins for ImageJ analysis software (41). Three independent stimulations were conducted using hemolymph from single animals. In each experiment, at least 40 individual cells were analyzed, and the maximum cell size, mean velocity and accumulated distance were measured. Statistical analysis was conducted using GraphPad Prism software (p<0.05).

## Results

### Assembly and Annotation of the Mussel Transcriptome and miRNome

A summary of the bioinformatics assessment used to analyse the transcriptome and miRNome data is presented in **Supplementary Figure 3**. The sequence origin, assembly, identification, and annotation results are shown in **Table 1**.

**Table 1:** Summary of the transcriptome and miRNome results.

Transcriptome		
Reads origin	Raw reads	Trimmed reads
Control 1	90,967,138	99.53%
Control 2	84,688,626	99.49%
Control 3	53,800,576	94.49%
Poly I:C 1	72,314,852	99.55%
Poly I:C 2	81,962,728	99.28%
Poly I:C 3	50,986,886	98.78%
Gluc 1	86,613,984	99.44%
Gluc 2	74,728,598	99.13%
Gluc 3	52,826,362	97.96%
LPS 1	84,290,784	99.44%
LPS 2	71,098,460	98.51%
LPS 3	52,584,202	98.92%
**Assembly**		
Transcripts	219,765	
Range transcript length	200- 16,164	
Average transcript length	493	
N50	539	
**Blast**		
Uniprot/SwissProt	37,841 (17.22%)	
Molluscs nucleotide NCBI	61,396 (27.94%)	
**GO analysis**		
Annotated transcripts	37,600 (17.12%)	
**KEGG analysis**		
Enzyme code assigned transcripts	11,301 (5.14%)	
**miRNome**		
**Reads origin**	**Raw reads**	**Trimmed reads**
Control	26,807,946	79.3%
Poly I:C	27,317,373	80.11%
β-glucans	28,387,740	76.3%
LPS	29,018,010	73.44%
**Assembly**		
Non-redundant miRNA clusters	5,175,567	
miRNA clusters - count over 52	69,995	
**Blast**		
Against miRBase v.22.1	1,550 (2.2%)	

High-throughput mRNA sequencing yielded an average of 71 million raw reads per sample. Over 97% of the raw reads successfully passed the quality control inspection in all the samples. A hemocyte transcriptome comprising 219,765 transcripts (average length 493 bp; N50 539 bp) was obtained after the assembly of all the mRNA reads. Blast2GO software was used to identify 17.22% of the transcripts by conducting BLASTx analysis against UniProt, and a local BLASTn was performed using CLC Workbench to annotate 27.92% of the transcripts. GO terms were assigned to 17.12% of the transcripts.

For miRNA, an average of 28 million raw reads was obtained from each sample. After filtering, over 95% of the raw reads passed the quality control inspection, and the reads in the 15-30 nucleotide range represented 73% to 80% of the total. A nonredundant miRNome composed of 5,175,567 putative miRNAs was obtained, 69,995 of these putative miRNAs had a TPM over 10, and 1,482 of these putative miRNAs matched a miRBase entry.

**Figure 1:**
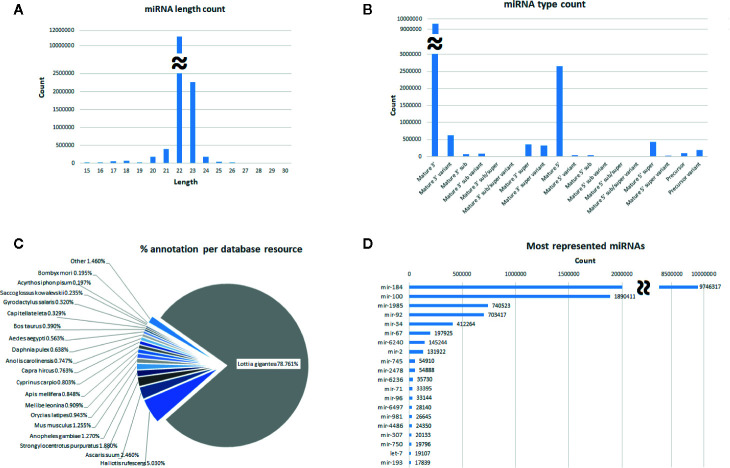
General description of the global miRNome. **(A)** miRNA distribution by length. **(B)** Distribution by miRNA subtype. **(C)** Pie chart showing the percentage of species hits after the miRBase annotation. **(D)** Distribution of the most represented miRNA families identified in the miRBase.

The general characteristics of this mussel miRNome are described in **Figure 1**. To identify the most abundant categories in the miRNome, miRNAs were counted. The length distribution shows that the majority of miRNAs are 21-23 nt in length. Regarding the miRNA subtypes, the mature miRNAs are the most represented subtype, followed by their variants. After identification of the miRNAs in the miRBbase, *Lottia gigantea* was the species with the most hits in our miRNome, followed by another mollusc (*Haliotis rufescens*), a nematode (*Ascaris suum*), and an insect (*Anopheles gambiae*). The most represented miRNA family was miR-184 (with nearly 10 million counts), followed by miR-100 (almost 2 million counts) and miR-1985 (over 700 thousand counts).

### Hemocytes Transcriptomic and miRNomic Response to PAMPs

**Figure 2:**
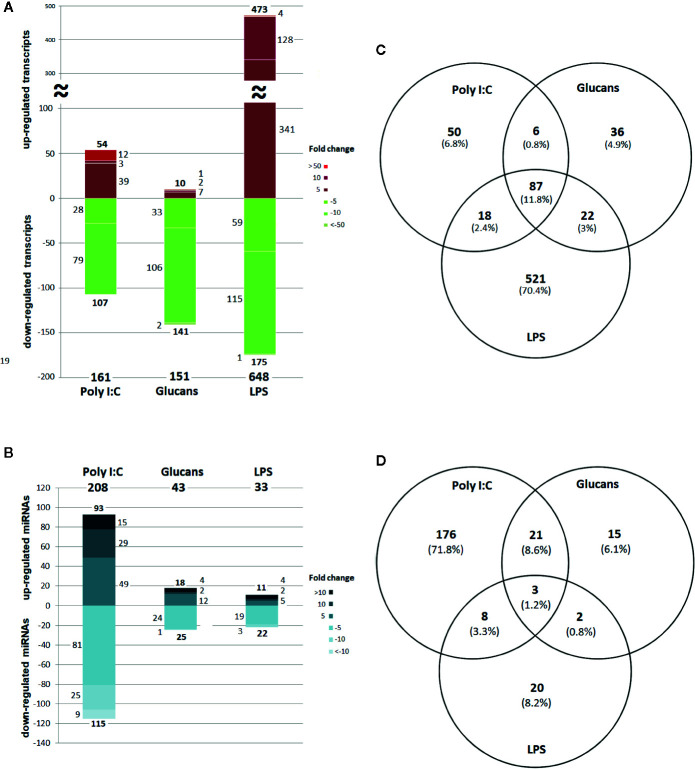
Distribution and Venn diagram of the differentially expressed RNAs in response to every stimulus compared to the control. **(A)** DEG (mRNA) distribution by fold change. **(B)** DE miRNA distribution by fold change. **(C)** Common and shared DEGs. **(D)** Common and shared DE miRNAs.

The range of expression of the differentially expressed genes (DEGs) in stimulated hemocytes compared to control hemocytes is shown in **Figure 2A**. Although poly I:C and β-glucans resulted in a similar number of DEGs, 161 DEGs (**Supplementary Table 1**) and 151 DEGs (**Supplementary Table 2**), respectively, LPS was the stimulus that elicited the highest response, with 648 DEGs (**Supplementary Table 3**). It is interesting to note that while the great majority of DEGs were upregulated in LPS-treated hemocytes, after stimulation with poly I:C and β-glucans, most of the DEGs were downregulated. On the other hand, the differentially expressed miRNAs (DE miRNAs) showed the opposite response (**Figure 2B**). The stimulus that triggered the strongest response was poly I:C, with 208 DE miRNAs (**Supplementary Table 4**), followed by β-glucans and LPS (with 43 DE miRNAs (**Supplementary Table 5**) and 33 DE miRNAs (**Supplementary Table 6**), respectively.

Considering all the DEGs associated with the three stimuli, 70.4% were modulated exclusively by LPS, 6.8% by poly I:C and 4.9% by β-glucans (**Figure 2C**). All these stimuli triggered a group of 87 common genes (11.8%), but only 18 of these common genes had informative annotations, which corresponded to: ADAT1 (tRNA-specific adenosine deaminase 1), ART2 (antisense to ribosomal RNA transcript protein 2), RRT15 (regulator of rDNA transcription protein 15), TAR1 (transcript antisense to ribosomal RNA protein 1) and histone H4. In addition, all these common genes are regulators of transcription and some are related to miRNA expression; some examples include NLRC3 (NLR family CARD domain containing 3), a negative regulator of the innate proinflammatory immune response; Cyp-like protein, involved in NADPH-dependent electron transport; senescence-associated protein, related to ageing and redox homeostasis; and CRP-I peptides and the conotoxin superfamily, small proteins with characteristics of bivalve AMPs (highly conserved pre-pro region, hypervariable mature peptide rich in cysteine residues and a net positive charge).

Regarding the common and exclusive DE miRNAs, after stimulation with the PAMPs, 72% corresponded exclusively to poly I:C, 6.1% to β-glucans and 8.2% to LPS (**Figure 2D**). Only 3 specific miRNAs were common to all PAMP responses: mir-71, mir-190, and mir-4171. Mir-71 upregulation is related to an increased lifespan through regulation of alg-1/Argonaute and DAF-16/FOXO in *Caenorhabditis elegans* (42, 43). Mir-190 is an efficient tumour suppressor that limits angiogenesis; the ZEB1-miR-190-SMAD2 interaction participates in TGF-β and VEGF regulation in humans (44, 45). Mir-4171 is a miRNA found in *Ciona intestinalis* with an unknown function. When we analysed the common and exclusive miRNAs according to miRNA families (**Supplementary Figure 2**), there were 16 families involved in the responses to all the PAMPs: let-7, mir-2, mir-8, mir-34, mir-67, mir-71, mir-87, mir-92, mir-100, mir-184, mir-190, mir-750, mir-981, mir-1985, mir-2478, and mir-4171. Many of these miRNAs are related to cancer, apoptosis, ageing and proliferation in mammals.

To validate the DEG results, the expression of IRG1, sacsin, histone H4, IRF2, CH24H, PGRP, MyD88a and MyD88c was studied by qPCR in different mussels; thus, this was not only a technical validation but also a biological validation. The statistical analysis showed a significant correlation between the fold changes obtained with both methodologies, confirming our expression results (**Supplementary Figure 1C**). The same approach was followed to validate the miRNome. The chosen miRNAs were let-7, mir-10, mir-71, mir-100, mir-125, mir-184 and mir-279, which also showed a significant correlation between the fold changes in qPCR and RNA-Seq (**Supplementary Figure 1D**).

### Processes and Pathways Related to the DEGs

**Figure 3:**
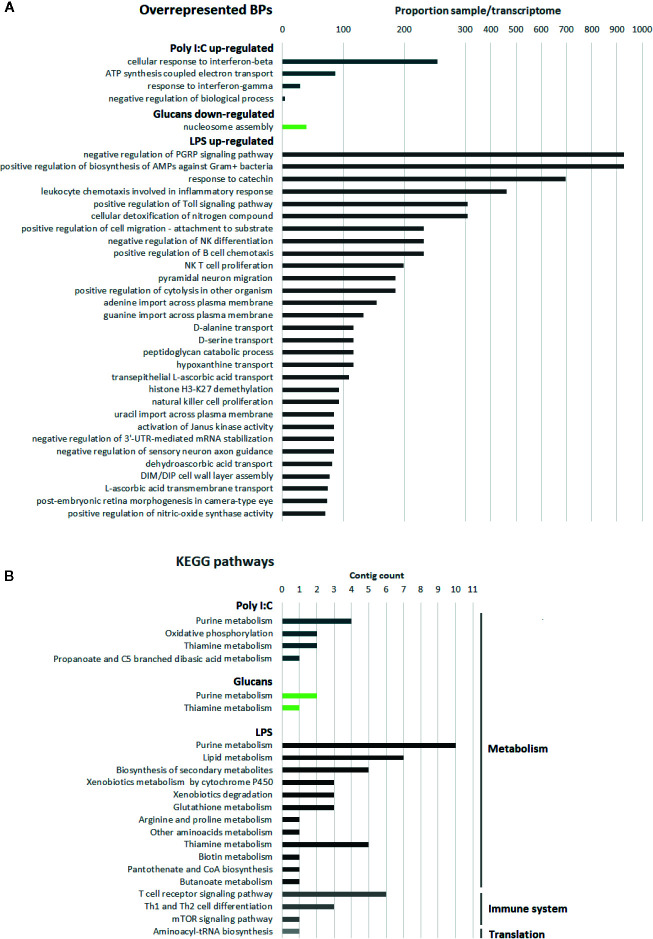
Description of the functional annotation for the DEGs. In blue, poly I:C; in green, β-glucans; in grey, LPS. **(A)** Enrichment analysis of the GO terms related to the DEGs for each stimulus. For LPS, only the top 30 overrepresented BPs are shown. **(B)** KEGG analysis of the exclusive DEGs for each stimulus.

The enrichment analysis of the GO terms related to the DEGs revealed 4, 1, and 203 overrepresented biological processes (BPs) for the responses to poly I:C, β-glucans and LPS, respectively (**Figure 3A**). The BPs involved in the response to poly I:C were related to the response to interferon, which is the main antiviral pathway. IRF2 (interferon regulatory factor 2) is the gene ascribed to these GO terms. Regarding β-glucans, the only overrepresented process was nucleosome assembly because of the four histone sequences that were downregulated in response to this stimulus. Last, LPS stimulation was associated with numerous processes related to the immune response but also processes related to cell migration, differentiation and proliferation, among others. However, we need to take into consideration that, due to the low percentage of mussel transcripts assigned to GO terms and that these terms are based on the function of their respective orthologous genes in vertebrates, these enrichment analyses could provide biased information.

The nonredundant DEGs and miRNA families that showed the highest changes in expression after poly I:C, β-glucan and LPS stimulation are presented in **Tables 2**–**4**, respectively (complete data in **Supplementary Tables 1**–**3**). Some of these most highly expressed genes are the common DEGs shared among all the stimuli presented in the previous section, and interestingly, all of these DEGs were downregulated. However, the upregulated genes were more specific for each stimulus. Regarding miRNA expression, common and exclusive transcripts were either up- or downregulated in response to all the stimuli.

**Table 2:** Top 30 non-redundant regulated DEGs and miRNA families in response to poly I:C.

Transcriptome			miRNome	
Transcript ID	FC	Description	miRNA	FC
Hem_contig_218378	1467.36	Genome polyprotein	**mir-67**	**44036.81**
Hem_contig_118857	984.23	Venom allergen 5	**mir-184**	**10794.95**
Hem_contig_82198	897.44	Trypsin-1	mir-993	647.52
Hem_contig_109302	294.65	Cytochrome c oxidase subunit 1 (COX1)	mir-1357	49.97
Hem_contig_204608	92.3	Cytochrome c oxidase subunit 3 (COX3)	**mir-71**	**29.76**
Hem_contig_119938	62.69	Cytochrome c oxidase subunit 2 (COX2)	mir-2001	13.77
Hem_contig_98645	58.11	Cytochrome b	mir-9	11.56
Hem_contig_23784	4.36	Sacsin	**mir-190**	**7.35**
Hem_contig_100181	4.27	Pre-mRNA cleavage complex 2 protein Pcf11	mir-263	7.29
Hem_contig_21324	3.61	E3 ubiquitin-protein ligase TRIM56	mir-22	7.21
Hem_contig_2525	3.33	Rapamycin-insensitive companion of mTOR (RICTOR)	mir-745	6.22
Hem_contig_28791	3.16	E3 SUMO-protein ligase RanBP2	mir-317	4.93
Hem_contig_62327	3.15	N-myc and STAT interactor	**let-7**	**4.80**
Hem_contig_36433	3.01	Interferon regulatory factor 2 (IRF2)	mir-10	4.61
Hem_contig_3804	2.96	Cis-aconitate decarboxylase (IRG1)	**mir-34**	**4.49**
Hem_contig_56299	2.9	Neurexin-2-alpha	mir-72	4.23
Hem_contig_43708	2.55	NFX1-type zinc finger-containing protein 1	mir-31	4.18
Hem_contig_145664	-5	Serine-threonine kinase receptor-associated protein	mir-307	-3.79
Hem_contig_53058	**-5.1**	**Transcript antisense to ribosomal RNA protein 1 (TAR1)**	**mir-100**	**-4.15**
Hem_contig_5136	**-5.1**	**Antisense to ribosomal RNA transcript protein 2 (ART2)**	**mir-4171**	**-4.20**
Hem_contig_4657	**-5.38**	**Protein NLRC3**	mir-1989	-4.92
Hem_contig_11657	-6	Elongation factor Tu 1	mir-1260	-6.17
Hem_contig_32700	**-6.7**6	**Histone H4**	**mir-2478**	**-6.74**
Hem_contig_196569	**-6.77**	**Senescence-associated protein**	**mir-34**	**-13.64**
Hem_contig_39941	**-7.47**	**tRNA-specific adenosine deaminase 1 (ADAT1)**	mir-365	-16.59
Hem_contig_28440	**-7.49**	**Conotoxin Superfamily**	mir-133	-21.30
Hem_contig_71914	**-7.67**	**Regulator of rDNA transcription protein 15 (RRT15)**	mir-184	-54.20
Hem_contig_35919	-8.66	Heat shock 70 DnaK (HSP70)	mir-193	-272.02
Hem_contig_36725	**-9.68**	**Cyp-like protein**	**mir-92**	**-805.96**
Hem_contig_162676	**-15.42**	**Cysteine-rich peptide CRP-I 2**	**mir-750**	**-1008.17**

FC, fold change; in the case of miRNAs FC is the mean of all the family members. In bold, common transcripts/miRNA families for all the stimuli. Highlighted in grey, exclusive transcripts/miRNA families after poly I:C stimulation.

**Table 3:** List of all nonredundant upregulated DEGs and miRNA families in response to β-glucans.

Transcriptome			miRNome	
Transcript ID	FC	Description	miRNA	FC
Hem_contig_73810	3.22	Protein turtle	**mir-2478**	**1494.25**
Hem_contig_38132	2.79	Target of Myb-like protein 2	**mir-8**	**833.24**
Hem_contig_32837	2.71	Cytochrome P450	mir-4171	442.19
Hem_contig_13359	2.63	Multidrug resistance protein 1 (CD243)	**mir-184**	**222.09**
Hem_contig_2938	2.47	Cholesterol 24-hydroxylase (CH24H)	**mir-87**	**7.18**
Hem_contig_12418	2.44	Solute carrier family 22 member 6-A	**mir-71**	**7.14**
			**let-7**	**3.62**
			**mir-981**	**3.23**
			mir-25	3.18
			**mir-190**	**2.35**
			**mir-750**	**2.18**
			mir-263	2.04
			**mir-92**	**2.35**
Hem_contig_35919	-4.72	Heat shock protein 70 DnaK (HSP70)	**mir-100**	**-1.22**
Hem_contig_53058	**-6.15**	**Transcript antisense to ribosomal RNA protein 1 (TAR1)**	**mir-8**	**-2.09**
Hem_contig_5136	**-6.26**	**Antisense to ribosomal RNA transcript protein 2 (ART2)**	**mir-2478**	**-2.11**
Hem_contig_196569	**-6.75**	**Senescence-associated protein**	mir-1175	-2.21
Hem_contig_36725	**-6.86**	**Cyp-like protein**	**mir-1985**	**-2.40**
Hem_contig_4657	**-7.03**	**NLRC3**	**mir-67**	**-2.43**
Hem_contig_32700	**-7.06**	**Histone H4**	**mir-2**	**-2.51**
Hem_contig_78138	-7.33	Histone H2B	mir-125	-2.60
Hem_contig_21480	**-7.5**	**Regulator of rDNA transcription protein 15 (RRT15)**	**mir-190**	**-2.61**
Hem_contig_120448	**-7.51**	**Cysteine-rich peptide CRP-I51**	**mir-92**	**-2.68**
Hem_contig_39941	**-7.53**	**tRNA-specific adenosine deaminase 1 (ADAT1)**	**mir-34**	**-2.77**
Hem_contig_28439	**-9.45**	**Conotoxin Superfamily**	mir-193	-3.40
Hem_contig_85889	-9.75	Small heat shock protein IbpA (HSP16)	mir-750	-4.65
Hem_contig_216322	-24.94	Histone H3.2	**mir-184**	**-3460.53**

FC, fold change; in the case of miRNAs, FC is the mean of all the family members. In bold, common transcripts/miRNAs families for all stimuli. Highlighted in grey, exclusive transcripts/miRNAs families after β-glucan stimulation.

**Table 4:** Top 30 nonredundant upregulated DEGs and all miRNA families in response to LPS.

Transcriptome			miRNome	
Transcript ID	FC	Description	miRNA	FC
Hem_contig_37773	65.44	Tubulin beta-5 chain	**mir-2478**	**3445.56**
Hem_contig_47522	34.45	Chorion peroxidase	**mir-2**	**534.83**
Hem_contig_42993	28.35	Tubulin alpha chain	**mir-184**	**482.74**
Hem_contig_1275	11.64	Autocrine proliferation repressor protein A	mir-252	22.93
Hem_contig_14066	11.44	Growth/differentiation factor 15 (GDF15)	MIR8175	9.42
Hem_contig_21389	10.14	Solute carrier family 7	**mir-71**	**7.34**
Hem_contig_14355	7.52	Sphingosine-1-phosphate lyase 1	**mir-4171**	**3.48**
Hem_contig_27520	7.07	ETS-related transcription factor Elf-5	**mir-26**	**3.18**
Hem_contig_37769	7.05	Hemicentin-2	**mir-87**	**2.99**
Hem_contig_102545	6.72	Temptin	**mir-67**	**2.28**
Hem_contig_92328	6.72	Scavenger receptor cysteine-rich type 1 protein M130 (CD163)	**mir-190**	**2.08**
Hem_contig_108916	6.63	Histone-lysine N-methyltransferase		
Hem_contig_14451	6.42	Mannose-binding protein C (MBL2)		
Hem_contig_5088	6.14	NFX1-type zinc finger-containing protein 1		
Hem_contig_13562	5.98	EGF-like protein		
Hem_contig_40924	5.97	Regulator of chromosome condensation		
Hem_contig_145664	-6.03	Serine-threonine kinase receptor-associated protein		
Hem_contig_5136	-6.25	**Antisense to ribosomal RNA transcript protein 2 (ART2)**		
Hem_contig_42579	-6.28	50S ribosomal protein L21	**mir-8**	**-2.03**
Hem_contig_4657	-6.65	**NLRC3**	bantam	-2.03
Hem_contig_36725	-7.05	**Cyp-like protein**	**mir-1985**	**-2.32**
Hem_contig_100242	-7.23	Histone H2A	mir-1984	-2.34
Hem_contig_21480	-7.28	**Regulator of rDNA transcription protein 15 (RRT15)**	**mir-100**	**-2.41**
Hem_contig_86385	-7.44	**Senescence-associated protein**	**mir-34**	**-2.65**
Hem_contig_32700	-7.6	**Histone H4**	**mir-981**	**-2.94**
Hem_contig_39941	-8.21	**tRNA-specific adenosine deaminase 1 (ADAT1)**	**let-7**	**-3.16**
Hem_contig_162676	-8.61	**Cysteine-rich peptide CRP-I 2**	**mir-92**	**-3.27**
Hem_contig_28439	-11.25	**Conotoxin Superfamily**	mir-10	-3.99
Hem_contig_216322	-16.56	Histone H3.2	**mir-67**	**-5.99**
Hem_contig_216855	-51.08	Peroxidasin	**mir-750**	**-6.91**

FC, fold change; in the case of miRNAs, FC is the mean of all the family members. In bold, common transcripts/miRNAs families for all stimuli. Highlighted in grey, exclusive transcripts/miRNAs families after LPS stimulation.

Considering the DEGs modulated by poly I:C stimulation, we observed different genes directly related to the immune response (TRIM56, RICTOR, IRF2, and NLRC3), oxidative stress (COX1, COX2, COX3, cytochrome b, and IRG1), and other cellular functions, such as cytoskeleton rearrangement (sacsin) (**Table 2**). On the other hand, immune- (NLRC3), and oxidative stress-related genes (cytochrome P450) were more limited after β-glucan treatment, although it is interesting to note the overexpression of CH24H, since lipid metabolism is intimately related to the immune response. The stimulation of hemocytes with LPS resulted in a high number of DEGs associated with immunity (GDF15, CD163, MBL2, NLRC3, TLR2, TRL4, TLR7, TLR13, peptidoglycan recognition protein 1 and 2 (PGRP1 and PGRP2), Caspase 3/7-3, mucosa-associated lymphoid tissue lymphoma translocation protein 1 (MALT1), MyD88, lipopolysaccharide-binding protein –LBP–, autophagy-related protein 2 homologue A (ATG2A), and NLRC3, among others) but also DEGs that function in oxidative stress (chorion peroxidase, glutathione S-transferase Mu 1 and 3 (GSTM1 and GSTM3), cytochrome P450 CYP12A2), lipid metabolism [sphingosine-1-phosphate lyase 1 (SGPL1), phthiocerol synthesis polyketide synthase type I PpsD, cholesterol 7-alpha-monooxygenase (CP7A1), acid sphingomyelinase-like phosphodiesterase 3b (ASM3B), ceramide kinase (CERK), and phospholipase D1 (PLD1)] and cell proliferation and differentiation (cyclin-F, ETS-related transcription factor Elf-5, tyrosine-protein kinase TEC, titin, hemicentin-1, etc.).

The KEGG pathways related to the DEGs that were unique to each stimulus (**Figure 3B**) included two common pathways: purine and thiamine metabolism. The pathways related to cellular metabolism were the most abundant in the KEGG analysis for all the stimuli. As expected, cellular metabolism changed after PAMP stimulation. Oxidative phosphorylation was exclusively observed after poly I:C stimulation, suggesting an increase in the energy demand in the hemocytes. In accordance with the results of the GO enrichment analyses, some KEGG pathways related to immune cell signaling and differentiation were exclusively observed after LPS stimulation. For this PAMP, the high representation of DEGs associated with lipid metabolism and response to oxidative stress was also clearly reflected in the KEGG pathways.

### Hemocytes Count, Phagocytosis, and ROS and NO Synthesis

**Figure 4:**
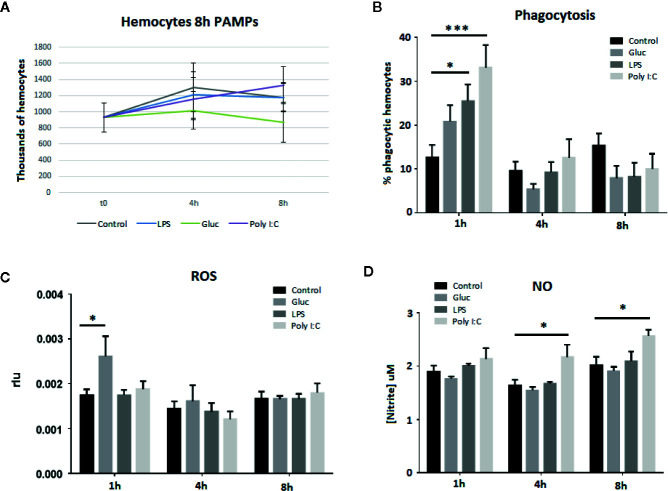
Hemocyte viability and functional assays after 8 h of treatment with the different stimuli. Bars show the standard error of the replicates, and asterisks indicate significant differences (*p-value < 0.05; ***p-value < 0.001). **(A)** Hemocyte viability. **(B)** Changes in phagocytic capacity. **(C)** ROS production. **(D)** NO production.

To confirm that the hemocytes were healthy throughout the experiment and that the transcriptomic results could indicate cell proliferation after PAMP treatment, hemocytes were counted after stimulation. The live hemocyte count results showed a mean of 928,000 cells/ml of hemolymph at the beginning of the experiment. This count remained relatively stable over time and no statistically significant increases or decreases in the hemocyte counts were observed in the control wells or in the poly I:C-, β-glucans-, or LPS-treated wells (**Figure 4A**).

Phagocytosis and respiratory burst are related to the cellular defense response but these processes could be also involved in hemocyte proliferation and death after PAMP treatment. The percentage of phagocytic cells increased 1 h after all PAMP treatments, although only LPS and poly I:C caused statistically significant increases (**Figure 4B**). Four hours after being exposed to the different stimuli, the number of phagocytic cells returned to control levels and remained at these levels until the end of the experiment. Regarding the respiratory burst, 1 h after β-glucans stimulation, a significant increase in ROS production was observed (**Figure 4C**). For the rest of the stimuli, ROS production remained almost constant over time, with no significant changes at the tested sampling points. This is in agreement with previous results, where *M. galloprovincialis* PMA-treated hemocytes stimulated with different PAMPs (poly I:C, zymosan, LPS, LTA, and CpG) only increased the ROS production after zymosan (β-glucan) stimulation (19). Similarly, NO production showed stable results over time, with the exception of poly I:C (**Figure 4D**). This stimulus was able to trigger significant NO production at 4 and 8 h after stimulation.

### Changes in the Hemocytes Morphology and Movement

Treatment with the PAMPS also influenced mussel hemocytes shape and movement, which are necessary for the response to immune stimuli. The analysis of cell size revealed that the morphology of hemocytes changed after their attachment on the plate and again after the treatments. Based on the measurement of a total of 390 control cells, the calculated mean size of the settled hemocytes at the beginning of the experiment was 29.6 µm (± 1 µm). The cell size significantly decreased in the control cells to 22.73 µm (± 1.6 µm) after 3 h of incubation in the plate at room temperature. For this experiment, 467 control cells were measured.

**Figure 5:**
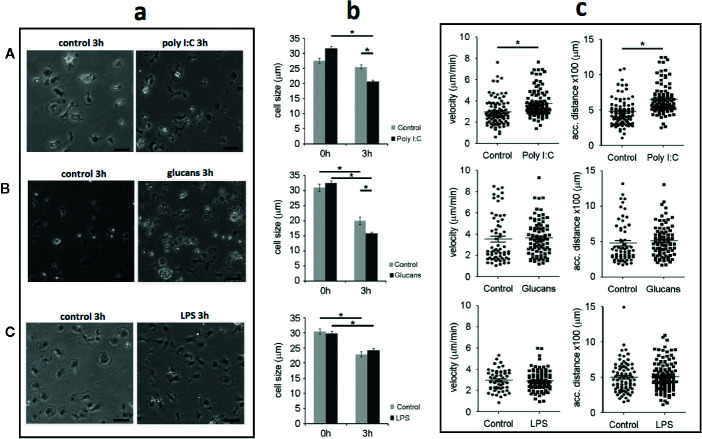
Description of the hemocyte size and movement characteristics. Note that for each stimulus, the control and treated hemocytes were harvested from the same animal. Asterisks show significant differences (*p-value < 0.05). **(A)** Morphology, size and movement after poly I:C stimulation. **(B)** Morphology, size and movement after β-glucans stimulation. **(C)** Morphology, size and movement after LPS stimulation. a. Morphological comparison between control and stimulated hemocytes. Scale bar: 50 µm. b. Distribution of cell size changes in response to each stimulus. c. Changes in the velocity and covered distance of hemocytes after stimulation.

The PAMPs also affected cell size and morphology. The cells treated with poly I:C and β-glucans showed a rounded morphology with a condensed cytoplasm after 3 h of stimulation (**Figures 5Aa, Ba**). At this time point, the size of the stimulated cells significantly decreased by 20% compared to that of the control cells. The cells treated with poly I:C were significantly smaller than the control cells (20.5 ± 0.4 µm for poly I:C-treated cells vs. 25.4 ± 0.7 µm for control cells) (**Figure 5Ab**). Treatment with β-glucans also significantly reduced the cell size from 19.8 ± 1.2 µm in the control cells to 15.7 ± 0.4 µm in the stimulated cells (**Figure 5Bb**). In contrast, LPS treatment did not modify cell morphology or size. After 3 h of stimulation, the cells settled on the plate surface (**Figure 5Ca**), and their cell size was similar to that of the control cells (24.2 ± 0.6 µm for LPS-treated cells vs 22.8 ± 0.9 µm for control cells) (**Figure 5Cb**).

Velocimetric parameters were also assessed, and the effect of PAMP stimulation was analyzed. Only treatment with poly I:C induced a significant increase in the mean hemocyte velocity after 3 h of stimulation. A total of 92 and 103 control and poly I:C-stimulated cells, respectively, were individually tracked for 3 h, and the final velocity was measured (**Figure 5Ac**). The velocity of the cells increased by 27% after poly I:C stimulation, changing from 2.9 ± 0.1 µm/min for the control cells to 3.7 ± 0.1 µm/min for the poly I:C-stimulated cells (**Figure 5Ac**). Compared with the control cells, the treatment of cells with β-glucans and LPS did not modify the mean velocity after 3 h of stimulation. The mean velocities of the cells stimulated with β-glucans or LPS were 3.5 ± 0.2 µm/min and 2.9 ± 0.1 µm/min, respectively (**Figures 5Bc, Cc**).

The total accumulated distance covered by stimulated hemocytes was also measured. Only the cells stimulated with poly I:C showed a significantly higher distance travelled than the control cells. The accumulated distance covered by the cells treated with poly I:C was significantly higher (32%) than that covered by the control cells (652.2 ± 21 µm for poly I:C-treated cells vs. 479.8 2 ± 21 µm for control cells) (**Figure 5Ac**). The accumulated distance of the cells treated with β-glucans and LPS was not different from that of the control cells (513 ± 23 µm for β-glucans and 509 ± 16 µm for LPS) (**Figures 5Bc, 5Cc**).

## Discussion

The interaction between the miRNome and transcriptome is key to understanding the physiological responses of all organisms. In vertebrates, it is expected that over 50% of expressed genes are subjected to regulation *via* miRNAs (46). In non-model species, the great majority of studies to date have been focused on either the gene expression profile or the miRNA profile, and studies about combinations of profiles are usually restricted to studies about specific mRNA-miRNA pairs. The understanding of the differential responses of the transcriptome and miRNome could help to explain the effective defense responses of mussels. In this work, we present, for the first time in bivalves, a combined study of DEGs and DE miRNAs after immune challenges of mussel hemocytes with different PAMPs.

The different PAMPs elicited specific response signatures in mussel immune cells. Poly I:C actively induced the production of miRNAs and modulation of genes mainly related to energy metabolism, oxidative stress, antiviral response and cell movement. LPS triggered only a small miRNomic response, but the transcriptomic response to LPS was the most intense of all the studied PAMPs; its gene signature was focused on bacterial recognition and elimination, detoxification and cell survival. The transcriptomic and miRNomic responses to β-glucans stimulation were well balanced but less obvious, although these responses seemed to be linked to cholesterol metabolism and cell survival.

Although, as expected, these stimuli showed very diverse transcriptomic and miRNomic responses, there were common processes activated by all the PAMPs. Genes related to protein transcription, metabolism, pathogen recognition and inflammation were common to all stimuli, as observed in previous works about stimulation of the innate immune system (47–49). Interestingly, all these common DEGs were downregulated in our results.

Immunometabolism is the regulated change in metabolism that is required to assist host defense and survival (50). Immunometabolism seems to play a central role in the mussel hemocytes response to PAMPs. One of the commonly downregulated genes was NLRC3. Recent research in humans has demonstrated that NLRC3, an almost unknown molecule, is an inhibitor of the inflammatory immune response, limits NF-κB activation and diminishes glycolysis and oxidative phosphorylation (51, 52). Therefore, the suppression of this gene in our transcriptome results may suggest that the inflammatory response and immunometabolic capacities were enhanced. Although the inhibition of NLRC3 expression was common to the three PAMPs, specific metabolic signatures were present for each PAMP.

In this regard, stimulation with poly I:C exclusively induced the expression of genes related to immunometabolism, including COX1, 2 and 3, and IRG1. Both groups of genes are related to energy metabolism and to the immune response through changes in mitochondrial metabolism (53) and the anti-inflammatory action of the IRG1 product itaconate (54–56), which was recently demonstrated in molluscs (57). Itaconate is known to exert an anti-inflammatory effect, limiting the activity of type I interferons (58), similar to IRF2 (59), which is also upregulated after poly I:C treatment and is known to promote IL-7 production and, consequently, hematopoiesis in vertebrates (60). Moreover, the antimicrobial role of itaconate has also been shown in mammals due to the inhibition of the glyoxylate cycle by this metabolite (55).

Because metabolic changes can also be related to cell differentiation, proliferation and taxis, and because some of the DEGs and DE miRNAs in response to PAMP stimulation could be directly related to this phenomenon, we analysed different aspects related to these cellular processes. Poly I:C, an interesting immunostimulatory molecule for mussels, increased the phagocytic activity of the hemocytes but also affected their morphology and enhanced their tactic behavior, as proven by functional assays. Sacsin, a key protein for the organization of the intermediate filament cytoskeleton (61) that exhibits antiviral properties (62), was highly represented and upregulated after poly I:C challenge. Sacsin could be involved not only in the antiviral response and phagocytosis but also in the morphological changes, increased velocity and increased distance travelled by hemocytes. miRNAs also seem to play relevant roles in these processes. miR-133 and miR-365, two miRNAs related to metabolism and differentiation in adipocytes and muscle (63, 64), were downregulated after poly I:C stimulation. The miRNomic response to poly I:C was stronger than that to the other PAMPs and inversely related to the transcriptomic response. This observation could be an indication that the antiviral response in mussel hemocytes would be biased towards miRNA transcription. Indeed, when Manila clams (*Ruditapes philippinarum*) were inoculated with the bacterial PAMPs LPS and peptidoglycan (PGN) and the viral stimulus poly I:C, lower gene modulation in the hepatopancreas was also observed in response to poly I:C (65). In contrast, the LPS miRNomic response is discrete when compared to the transcriptomic response, that it is by far the highest response elicited by the three PAMPs. The opposite trends between mRNAs and miRNAs could be due to the fact that miRNAs mainly act as key mechanisms of post-transcriptional gene silencing by degrading the target mRNAs (22).

A clear hemocyte immune response was observed after LPS stimulation. This PAMP efficiently triggered the expression of many non-self-recognition and immune-response genes, such as TLRs, PGRPs, LBP and lectins (mantle gene, galaxin, and perlucin), among others. PGRP and different lectins were also induced by LPS in the hepatopancreas of *R. philippinarum*, although the TLR signalling pathway seemed to be unaffected (65). Interestingly, a high number of complement-related genes was induced in that case (65). The pathways of PAMP recognition have been proposed as driving mechanisms of innate immune memory (66, 67). In the absence of danger, the response to PAMPs promotes homeostasis and regeneration rather than inflammation (68). Although inflammation is considered a vertebrate response, an inflammatory-like phenomenon has also been reported in invertebrates (69). In our results, this inflammatory-like response seems to be well controlled after LPS stimulation; neither ROS nor NO production were detected as early as 1 h after LPS stimulation. This result could be explained by the transcriptomic analysis; there are many upregulated genes, such as peroxidase and glutathione S-transferases, related to detoxification processes (reactive oxygen species metabolic process, glutathione transferase activity, peroxidase activity, and detoxification of nitrogen compounds), which could be indicative of a protective and regenerative state of hemocytes after LPS stimulation. Moreover, one of the most highly overexpressed genes after LPS challenge was GDF15, which was previously identified as a metabolic regulator involved in tolerance to LPS-induced sepsis in vertebrates through the alteration of lipid and glucose metabolism to avoid inflammatory damage (70, 71).

In addition, LPS promotes phagocytosis, which is consistent with the immunometabolic changes after this challenge. Two transcripts annotated as tubulin, namely, tubulin beta-5 chain and tubulin alpha chain, were overexpressed by 65.44- and 28.35-fold, respectively, after LPS stimulation. Overexpression of transcripts annotated as tubulin alpha-3 chain and dynein heavy chain were also observed in Manila clams stimulated with LPS or PGN (65). Tubulin chains constitute the cell microtubules, which are essential for phagocytosis, a pivotal mechanism both for the clearance of pathogens and the removal of apoptotic cells and debris to maintain homeostatic conditions (72). The presence of butyrate and propionate could enhance the phagocytic capacities of hemocytes (73) to eliminate pathogens and damaged cells. The metabolism of these two compounds is upregulated after LPS and poly I:C stimulation, respectively, suggesting an increase in phagocytosis with these stimuli.

On the other hand, cell proliferation and differentiation processes were found to be enriched after LPS stimulation, as well as one KEGG pathway related to immune cell differentiation, with included genes such as elf-5 (74), cyclin-F (75), titin (76), hemicentin-1 (77) and TEC (78), among others. Despite these data, the hemocytes counts did not follow a clear increasing trend in our experiments.

LPS also triggers the expression of cholesterol 7 alpha-monooxygenase, an important enzyme for cholesterol homeostasis. Cholesterol metabolism is key in immune defense and innate immune memory in vertebrates (79, 80). In our results, not only LPS but also β-glucans were shown to activate cholesterol metabolism. The stimulation of mussel hemocytes with β-glucans upregulated cholesterol 24-hydroxylase, which converts cholesterol into 24S-hydroxycholesterol and, to a lesser extent, 25-hydroxycholesterol. Hydroxylated forms of cholesterol (oxysterols) are considered important regulators of immune function, promoting an anti-inflammatory state (81) and exerting antiviral activities in vertebrates (82–84). It seems that although β-glucans elicited the most modest response at the transcriptomic and miRNomic levels, its immunomodulatory and cell protective potential should be further studied in molluscs, as it could be as strong as in fish and mammal species (85, 86).

Our results suggest that miRNAs may play a role in modulating the transcription programmes of hemocytes in response to these stimuli, especially to poly I:C. It is already known that in addition to their regulatory function in gene expression, miRNAs could enhance immunostimulation through PRRs and function as cell-to-cell signalling molecules (87, 88). Certain miRNAs possess an immunostimulatory motif (UGUGU) and/or serve as TLR7/8 ligands, and some of them are represented in our results: let-7, one of the most highly represented miRNAs in our results for all stimuli; miR-34, also common for all the PAMPs; and miR-133, differentially downregulated after poly I:C challenge. All these miRNAs are related to innate immunity, development, ageing and senescence in molluscs (89), insects (90, 91), fish (92), and mammals, including humans (93–97).

Ageing and senescence seem to play crucial roles in both the miRNomic and transcriptomic immune responses to PAMPs *in vitro*. This is consistent with recent research topics related to immunosenescence/inflamm-ageing (98), the name given to the adaptation to chronic insults that is necessary for adequate responses to known antigens. In this regard, miR-71 and miR-34 are important miRNAs related to ageing that were up- and downregulated, respectively, in our results; miR-71 overexpression promotes longevity and decreases age-dependent miRNA expression (99), and miR-34 repression promotes cell division (95). At the transcriptomic level, senescence-associated protein (SAP) was downregulated after all the challenges. This protein is poorly characterized in animals and is known to regulate leaf senescence during plant development through redox homeostasis (increased production of ROS). In plants, the upregulation of SAP is correlated with accelerated ageing (100), and its downregulation in humans is related to a proliferative state in cancer (101). These facts suggest the regulation of ROS production and a proliferative state of hemocytes after PAMP stimulation when SAP is downregulated. The functional results obtained (low ROS levels and stable hemocyte count despite the activation of phagocytosis) support this hypothesis.

The downregulated SAP has very high sequence similarity to RRT15 and ART2, showing a possible function of this protein in the modulation of rDNA transcription (102) and consequently in protein levels. This may indicate that ribosome biogenesis plays roles in senescence in mussels, as is known in other species (103). The excess of synthesized proteins is another characteristic of senescent cells. Senescence activates the transcription factor FOXO to induce E3 ubiquitin ligase production, which reorganizes the proteasome of cells undergoing senescence (104). In our results, FOXO expression was not altered in response to any of the stimuli, and the few upregulated ubiquitin ligases were always related to the innate immune response (TRIM56 and MIB2) and to cell proliferation (TRIM33 and MYCB2). The only downregulated ubiquitin ligase (synoviolin) is related to an antiapoptotic state. This observation confirms, once more, the proliferating phenotype of hemocytes, the promotion of cell death of damaged cells, and the lack of cellular maintenance through senescence, after PAMP stimulation.

In summary, although all the PAMPs showed specific responses, all of these responses shared common mRNAs and miRNAs. The control of the inflammatory-like response and cellular ageing seem to be important processes after immune challenge in mussel hemocytes. Immunostimulation through PRRs has a controlled proinflammatory component in the absence of microbial components (105, 106). This non-inflammatory response can also be observed in our results. Inflammation-related molecules were not observed in the transcriptome or in the miRNome described in this manuscript. In contrast, genes related to an anti-inflammatory state were significantly modulated after hemocytes stimulation. In addition, apoptosis, in contrast to senescence and pyroptosis, induces an immune response for the control of inflammation and tissue repair (106), highlighting the anti-inflammatory phenotype of hemocytes after PAMP stimulation. Therefore, although hemocyte proliferation cannot be demonstrated by these results, transcriptome data could suggest that proliferation occurs. More investigations will be needed to further address this question.

## Data Availability Statement

The datasets presented in this study can be found in online repositories. The names of the repository/repositories and accession number(s) can be found in the article/**Supplementary Material**.

## Ethics Statement

The Mediterranean mussel, *M. galloprovincialis*, is not considered an endangered or protected species in any international species catalogue, including the Convention on International Trade in Endangered Species (CITES) list (www.cites.org). *M. galloprovincialis* is not included in the European Union (EU) regulation to work with research animals by the Directive 2010/63/EU. Therefore, no specific authorization is required to work with mussels.

## Author Contributions

BN and AF obtained the funding and designed and supervised the experiments. RM, MR-C, and AR conducted the laboratory experiments. RM, PP, UR, BN, and AF analyzed the transcriptome and miRNA data. RM, PP, AR, UR, AF, and BN prepared the manuscript. All authors contributed to the article and approved the submitted version.

## Funding

This research was funded by the Spanish AEI/EU-FEDER (RTI2018-095997-B-I00); the European Regional Development Fund (ERDF) Interreg V Spain—Portugal (0474_BLUEBIOLAB); the Spanish Ministerio de Economía y Competitividad, project AGL2015-65705-R; Consellería de Economía, Emprego e Industria—GAIN, Xunta de Galicia, project IN607B 2019/01 and EU H2020, project VIVALDI (678589). MR-C acknowledges additional funding from the Spanish Ministerio de Economía y Competitividad for her predoctoral contract, BES-2016-076302. PP wishes to thank the Axencia Galega de Innovación (GAIN, Xunta de Galicia) for her postdoctoral contract, IN606B−2018/010.

## Conflict of Interest

The authors declare that the research was conducted in the absence of any commercial or financial relationships that could be construed as a potential conflict of interest.
