# Supplementary material for: Stimulation of Mytilus galloprovincialis Hemocytes With Different Immune Challenges Induces Differential Transcriptomic, miRNomic, and Functional Responses
Source: Front Immunol. 2020 Dec 17;11:606102. doi: 10.3389/fimmu.2020.606102 (PMC7773633; doi:10.3389/fimmu.2020.606102)
Supplement: Supplementary file 2 [file DataSheet_1.docx]

Supplementary Material

# Supplementary Figures


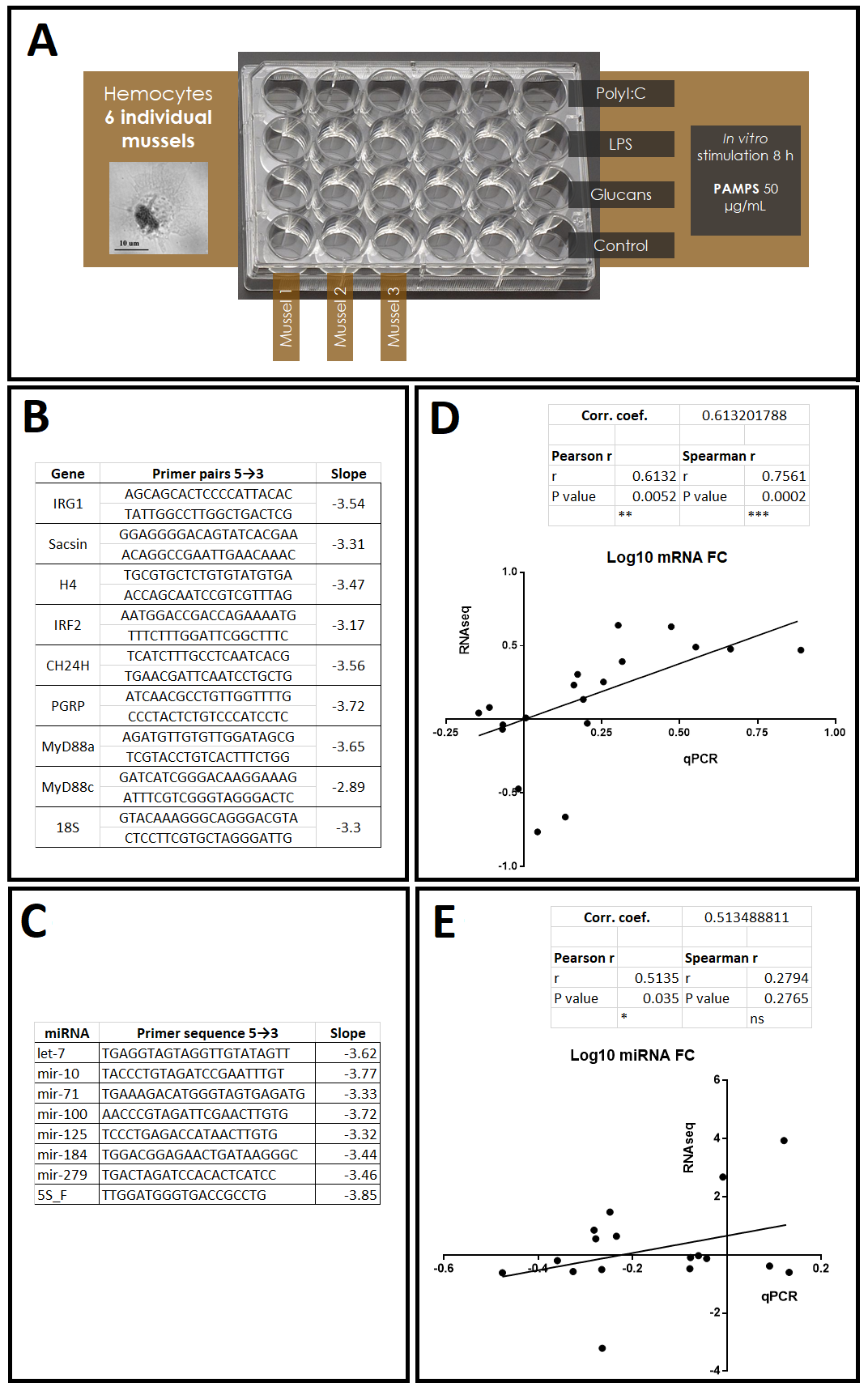


# Supplementary Figure 1. Validation experiment and analysis of the differentially expressed mRNAs and miRNAs. A) Experimental design with individual mussels to validate the transcriptome and miRNome expression results. B) Primer pairs used for mRNA expression validation. The slope to calculate primer pairs efficiency is indicated. C) Mean CTs and standard deviation of mRNA genes used in the validation. D) Primer pairs used for miRNA expression validation. The slope to calculate primer pairs efficiency is indicated. The reverse primer was the universal primer included in the miScript SYBR Green PCR Kit. E) Mean CTs and standard deviation of miRNAs used in the validation. F) Linear regression and statistical correlation of RNA-Seq and qPCR results for the chosen genes and conditions. Both parametric and non-parametric analyses showed significant correlation. G) Linear regression and statistical correlation of RNA-Seq and qPCR results for the chosen miRNAs and conditions. Parametric analysis showed significant correlation.


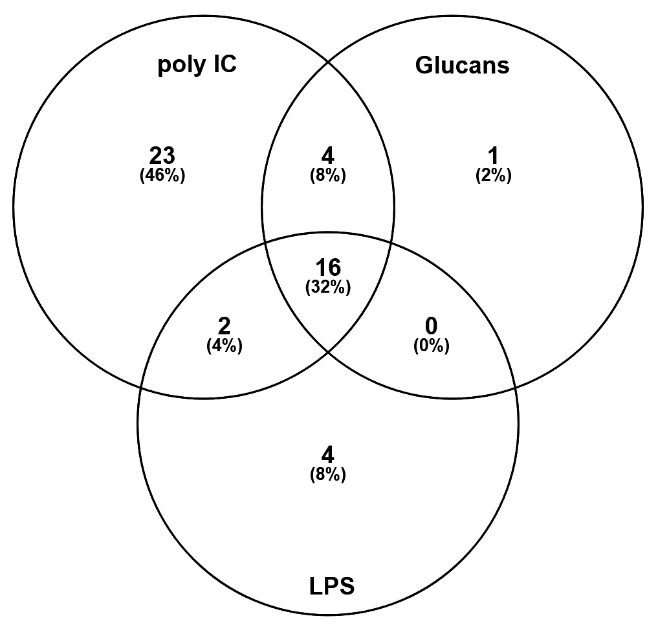


| **Common** |  | **Poly I:C excl** | | **Gluc excl** |  | **LPS excl** |  |
| --- | --- | --- | --- | --- | --- | --- | --- |
| let-7 |  | mir-1260 |  | mir-25 |  | mir-252 |  |
| mir-100 |  | mir-133 |  |  |  | MIR8175 |  |
| mir-184 |  | mir-1357 |  |  |  | mir-26 |  |
| mir-190 |  | mir-1989 |  |  |  | mir-1984 |  |
| mir-1985 |  | mir-1994 |  |  |  |  |  |
| mir-2 |  | mir-1996 |  |  |  |  |  |
| mir-2478 |  | mir-2001 |  |  |  |  |  |
| mir-34 |  | mir-22 |  |  |  |  |  |
| mir-4171 |  | mir-278 |  |  |  |  |  |
| mir-67 |  | mir-279 |  |  |  |  |  |
| mir-71 |  | mir-281 |  |  |  |  |  |
| mir-750 |  | mir-307 |  | **Poly I:C and Gluc** | | **Poly I:C and LPS** | |
| mir-8 |  | mir-31 |  | mir-263 |  | mir-10 |  |
| mir-87 |  | mir-317 |  | mir-1175 |  | bantam |  |
| mir-92 |  | mir-365 |  | mir-125 |  |  |  |
| mir-981 |  | mir-4486 |  | mir-193 |  |  |  |
|  |  | mir-72 |  |  |  |  |  |
|  |  | mir-745 |  |  |  |  |  |
|  |  | mir-7975 |  |  |  |  |  |
|  |  | mir-9 |  |  |  |  |  |
|  |  | mir-96 |  |  |  |  |  |
|  |  | mir-99 |  |  |  |  |  |
|  |  | mir-993 |  |  |  |  |  |

**Supplementary Figure 2. Distribution of the common and exclusive miRNA families after PAMPs stimulation of hemocytes.**


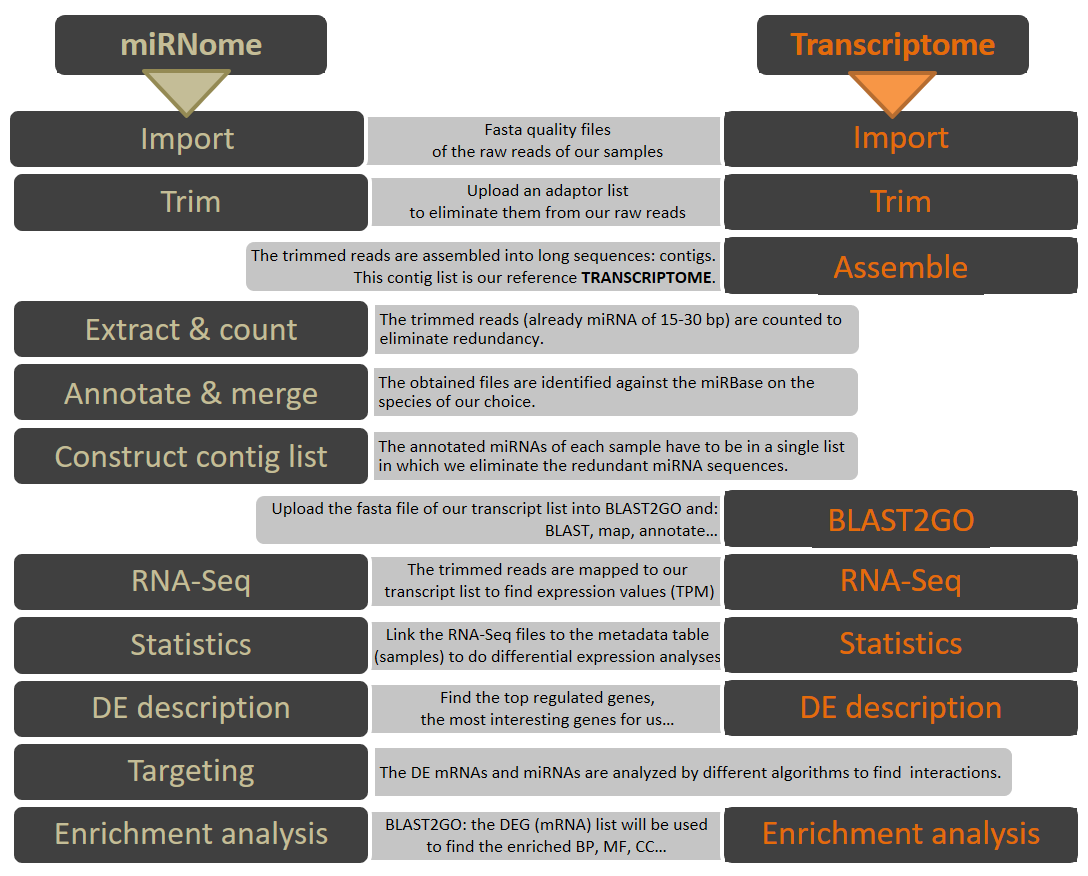


**Supplementary Figure 3. Bioinformatics assessment to analyse transcriptome and miRNome data.**
